# Aetiologies and clinical presentation of central nervous system infections in Vietnamese patients: a prospective study

**DOI:** 10.1038/s41598-022-23007-0

**Published:** 2022-10-27

**Authors:** Julian Justin Gabor, Chu Xuan Anh, Bui Tien Sy, Phan Quoc Hoan, Dao Thanh Quyen, Nguyen Trong The, Salih Kuk, Peter G. Kremsner, Christian G. Meyer, Le Huu Song, Thirumalaisamy P. Velavan

**Affiliations:** 1 Institute of Tropical Medicine, University of Tübingen, Wilhelmstrasse 27, 72074 Tübingen, Germany; 2 Vietnamese - German Centre for Medical Research (VG-CARE), Hanoi, Vietnam; 3 Department of Infectious Diseases, 108 Military Central Hospital, Hanoi, Vietnam; 4 Department of Microbiology, 108 Military Central Hospital, Hanoi, Vietnam; 5 Department of Molecular Biology, 108 Military Central Hospital, Hanoi, Vietnam; 6 Centre de Recherches Medicales de Lambarene, Lambaréné, Gabon

**Keywords:** Clinical microbiology, Infectious-disease diagnostics, Virology, Microbiology, Molecular biology, Diseases, Medical research, Molecular medicine

## Abstract

Knowledge of the clinical presentation of central nervous system (CNS) infections and the causative pathogens is crucial for appropriate diagnosis and rapid initiation of appropriate treatment to prevent severe neurological sequelae. The aim of this study is to understand the aetiology of CNS infections based on the clinical presentation of Vietnamese patients. A prospective hospital-based cohort study was conducted between May 2014 and May 2017. We screened 137 patients with clinically suspected CNS infection for fungal, bacterial and viral pathogens using their cerebrospinal fluid (CSF) and blood cultures. In addition, DNA or RNA extracted from CSF samples were subjected to nucleic acid testing (NAT) with a selective panel of bacterial, viral and fungal pathogens. At least one pathogen could be detected in 41% (n = 56) of the patients. The main pathogens causing CNS infections were *Streptococcus suis* (n = 16; 12%) and *Neisseria meningitidis* (n = 9; 7%), followed by Herpes simplex virus 1/2 (n = 4; 3%) and *Klebsiella pneumoniae* (n = 4; 3%). Other pathogens were only identified in a few cases. Patients with bacterial CNS infections were significantly older, had a worse outcome, a lower Glasgow Coma Scale (GCS), a higher rate of speech impairment and neck stiffness than patients with viral or tuberculous CNS infections. In northern Vietnam, adults are mostly affected by bacterial CNS infections, which have a severe clinical course and worse outcomes compared to viral or tuberculous CNS infections. Clinicians should be aware of the regional occurrence of pathogens to initiate rapid and appropriate diagnosis and treatment.

## Introduction

Infections of the central nervous system (CNS) like encephalitis, meningitis or meningoencephalitis are a frequent cause of hospitalization in Southeast Asia. In a large number of cases no causative pathogen can be determined^1^. Although treatment options are available for viral, bacterial and fungal infections, including vaccinations for a few, a large number of remain in which the aetiology of the CNS infection is not clear^2,3^. Such undifferentiated aetiologies can be diagnosed by introducing appropriate and easy to apply diagnostic protocols, especially in low and middle-income countries (LMICs).

The use of cerebrospinal fluid (CSF) in CNS infection diagnostics is inevitable. The cell counts, glucose and protein levels in CSF serve as markers and predictors to differentiate aseptic, bacterial and eosinophilic meningitis. For accurate determination of the causative pathogen, CSF and/or blood cultures are needed, however they are time consuming. The molecular diagnostic methodologies such as PCR and real-time PCR offer a quick alternative and aid in timely diagnosis.

Infections of the CNS such as encephalitis, meningitis and/or meningoencephalitis are common causes for hospitalization in Southeast Asia. Vietnam is an LMIC where the causative pathogens vary in time and space, especially in large urbanized cities^3^. The causative agents of CNS infections especially in Northern Vietnam are poorly investigated and often lack appropriate microbiological confirmation. An earlier prospective study concluded on the need of improved diagnostics for meningoencephalitis syndromes^4^.

Our aim was to investigate and document the actual pattern of the various pathogens causing CNS infection in Vietnamese patients suffering from meningitis and encephalitis. In this context, we recruited and screened hospitalized patients with CNS infections based on their clinical presentation. First, we screened for causative pathogens using blood and CSF cultures. In addition, PCR and real-time PCR methods were developed for a panel of 19 distinct potentially causative bacterial, viral and fungal pathogens. We correlated the observed clinical phenotypic presentation with our experimental results.

## Results

### CNS infections

In 137 included patients, we were able to determine at least one pathogen in 56 cases (41%). Of these 56 cases, 49 (36%) were diagnosed either by CSF culture or CSF PCR (Table1), the remaining 7 (5%) pathogens were determined by blood culture (Table 2). The main causes of CNS infections were *Streptococcus suis* (n = 16; 12%) and *Neisseria meningitidis* (n = 9; 7%), followed by Herpes simplex virus 1/2 (n = 4; 3%) and *Klebsiella pneumoniae* (n = 4; 3%). Three patients were found to be co-infected with either *Listeria monocytogenes* plus EBV, *K. pneumoniae* plus *N. meningitidis* and a rickettsial infection plus *M. tuberculosis*. In addition, one case each of *Burkholderia pseudomallei*, *Stenotrophomonas maltophilia*, *A. baumannii*, *Escherichia coli*, *S. suis* and two cases of *S. aureus* were identified in blood cultures. There was one suspected eosinophilic meningitis case (≥ 10/µl eosinophilic leukocytes), but CSF RT-PCR showed *S. suis* as the causative pathogen. All CSF samples were also tested by PCR or real-time PCR for *Orientia tsutsugamushi,* Cytomegalovirus, Parvovirus B19*, Haemophilus influenza b, Leptospira interrogans,* dengue virus 1, 2, 3, and measles virus*,* but none of these pathogens could be detected in the available CSF samples.

**Table 1:** CNS infections detected by CSF culture and/or by nucleic acid testing.

Pathogens	N = 49 (%)	CSF culture	CSF Nucleic acid testing	CSF culture and Nucleic acid testing
*Aerococcus viridans*	1 (1%)	1	NA	NA
*C. neoformans*	1 (1%)	1	0	0
*E. coli*	1 (1%)	1	NA	NA
*E. faecalis*	1 (1%)	1	NA	NA
*EBV*^*1*^	1 (1%)	NA	1	NA
*HSV*	4 (3%)	NA	4	NA
*Haemophilus parainfluenzae*	1 (1%)	1	NA	NA
*K. pneumoniae*^*2*^	4 (3%)	0	1	3
*M. tuberculosis*^*3*^	2 (2%)	0	2	0
*N. meningitidis*^*2*^	9 (7%)	2	6	1
*P. aeruginosa*	1 (1%)	0	1	0
*L. monocytogenes*^*1*^	1 (1%)	0	0	1
*Rickettsia genus*^*3*^	3 (2%)	NA	3	NA
*S. aureus*	1 (1%)	0	0	1
*S. pneumoniae*	3 (2%)	0	3	0
*S. suis*	15 (11%)	1	6	8

^1,2,3^Pathogens occurred as co-infections with each other; NA: diagnostic procedure not available for this pathogen.

**Table 2:** CNS infections detected by blood culture.

Pathogens	N = 7 (%)
*Burkholderia pseudomallei*	1 (1%)
*Stenotrophomonas maltophilia*	1 (1%)
*A. baumannii*	1 (1%)
*E. coli*	1 (1%)
*S. suis*	1 (1%)
*S. aureus*	2 (2%)

### CNS infections and clinical outcome

Of the 137 cases examined, 25 cases with suspected CNS infection were excluded (Table 3) because there was insufficient information on the characteristics of the CSF and no pathogen could be detected. In one of the 25 excluded cases, one confirmed case of fungal infection with *C. neoformans* was excluded as it did not meet the criteria for stratification. The remaining 112 cases were categorized based on CSF characteristics or the pathogen detected in microbiological or molecular testing and were classified as either bacterial meningitis (BI: n = 57, 51%), aseptic/viral encephalitis/meningitis group (VI: n = 50, 45%) or tuberculosis meningitis (TI: n = 5, 4%) (Table 3). 89 patients (79%) were male in the stratified groups with distinct CNS infections (Table 3).

**Table 3:** CNS patient characteristics and CNS infection aetiology.

	Bacterial infection (BI) n (IQR) or (%)		Viral/aseptic infection (VI) n (IQR) or (%)		M. tuberculosis infection (TI) n (IQR) or (%)		*p-values*
CSF Leukocytes/µl	n = 50^1^	1125 (233–4920)	n = 50^2^	94 (44.5–212.5)	n = 5	50 (36–560)	BI vs. VI = < .0001* BI vs. TI = 0.007* VI vs. TI = NS*
CSF-Polymorphonuclear (%)		90 (80–97)		8 (3–12)		32 (2.5–46)	BI vs. VI = < .0001* BI vs. TI = . 0007* VI vs. TI = . NS*
CSF Lymphocytes (%)		10 (3–20)		92 (88–97)		68 (54–97.5)	BI vs. VI = < .0001* BI vs. TI = .0005* VI vs. TI = NS*
CSF Protein (g/L)		3.5 (1.59–5.96)		0,91 (0.58–1.34)		2,45 (1.32–4.4)	BI vs. VI = < .0001* BI vs. TI = NS* VI vs. TI = 0.02*
Glucose (mg/dL)		29.7 (3.15–56.26)		64,87 (53.15–81.53)		32,4 (18–54)	BI vs.VI = < .0001* BI vs. TI = NS* VI vs. TI = 0.004*
Outcome at discharge	n = 54		n = 49		n = 5		
Recovery		43 (80%)		46 (94%)		5 (100%)	BI vs. VI = 0.035^+^ BI vs. TI = NS^+^ VI vs. TI = NS^+^
Partial recovery		5 (9%)		1 (2%)		0 (0%)	BI vs. VI = NS^+^ BI vs. TI = NS^+^ VI vs. TI = NS^+^
Fatalities		6 (11%)		2 (4%)		0 (0%)	BI vs. VI = NS^+^ BI vs. TI = NS^+^ VI vs, TI I = NS^+^
Gender	n = 57	Male:48; Female:9	n = 50	Male:37; Female:13	n = 5	Male:4; Female:1	
Age, years	n = 57	54 (31.5–62.5)	n = 50	36.5 (28–57)	n = 5	31 (20.5–61.5)	BI vs. VI = 0.025* BI vs. TI = NS* VI vs. TI = NS*
Days of illness before admission	n = 57	3 (2–5)	n = 50	4 (2–8)	n = 5	10 (5.5–10)	BI vs. VI = 0.0045* BI vs. TI = 0.0045* VI vs. TI = NS*
History of fever	n = 56	56 (100%)	n = 50	48 (96%)	n = 5	5 (100%)	BI/VI = .13^+^ BI/TI = .0^+^ VI/TI = .65^+^
Max. temperature (hospitalization period)	n = 56	39 (39–40)°C	n = 48	39 (38–39.5)°C	n = 5	38.6 (38.2–39)°C	BI vs. VI = 0.004* BI vs. TI = 0.032* VI vs. TI = . NS*
Days from admission to lumbar puncture	n = 56	0 (0–0.75)	n = 50	0 (0–0.25)	n = 4	0	BI vs. VI = NS* BI vs. TI = NS* VI vs. TI = NS*
Vomiting	n = 54	18 (33%)	n = 50	22 (44%)	n = 5	1 (20%)	BI vs. VI = NS^+^ BI vs. TI = NS^+^ VI vs. TI = NS^+^
Altered level of consciousness	n = 55	26 (47%)	n = 50	15 (30%)	n = 5	1 (20%)	BI vs. VI = NS^+^ BI vs. TI = NS^+^ VI vs. TI = NS^+^
Glasgow Coma Scale (GCS)	n = 56	13 (12–15)	n = 50	15 (13–15)	n = 5	15 (14–15)	BI vs. VI = 0.003* BI vs. TI = NS* VI vs. TI = NS*
Speech disorders	n = 54	30 (56%)	n = 50	15 (30%)	n = 5	0 (0%)	BI vs. VI = 0.008^+^ BI vs. TI = NS^+^ VI vs. TI = NS^+^
Neck stiffness	n = 54	53 (98%)	n = 50	36 (72%)	n = 5	3 (60%)	BI vs. VI = .0009^+^ BI vs. TI = .0018^+^ VI vs. TI = NS^+^
Cranial nerve palsy	n = 56	1 (2%)	n = 49	2 (4%)	n = 5	1 (20%)	BI vs. VI = NS^+^ BI vs. TI = .028^+^ VI vs. TI = NS^+^
Headache	n = 54	53 (98%)	n = 50	50 (100%)	n = 5	5 (100%)	BI vs. VI = NS^+^ BI vs. TI = NS^+^ VI vs. TI = NS^+^
Sensitivity disorder	n = 54	4 (7%)	n = 49	1 (2%)	n = 5	0 (0%)	BI vs. VI = NS^+^ BI vs. TI = NS^+^ VI vs. TI = NS^+^
Limb paresis	n = 54	4 (7%)	n = 49	0 (0%)	n = 5	0 (0%)	BI vs. VI = 0.052^+^ BI vs. TI = NS^+^ VI vs. TI = NS^+^
Convulsions	n = 53	2 (4%)	n = 49	1 (2%)	n = 5	0 (0%)	BI vs. VI = NS^+^ BI vs. TI = NS^+^ VI vs. TI = NS^+^

Data are presented in median values with respective interquartile range (IQR) and in percentages (%), where appropriate; *BI* bacterial infection, *VI* viral infection, *TI* *M.tuberculosis* infection; Significant *p-*values are shown; NS: non-significant.

*Indicates non-parametric Mann–Whitney–Wilcoxon rank sum test for continuous variables.

^+^Indicates chi-square test for comparison of the distribution of categorical variables (Yes/No).

^1^Cases were excluded if no CSF parameters were available (WBC, leukocytes, lymphocytes, protein, glucose).

^2^Co-infection of EBV and *L. monocytogenes* not included, Cases were excluded if no CSF parameters were available (WBC, leukocytes, lymphocytes, protein, glucose).

Of the 137 patients examined, an outcome report was available for 130 patients at discharge. Of these, 110 (85%) patients recovered completely, 9 (7%) patients recovered partially with neurological sequelae at discharge and 11 (9%) patients died during hospitalization. In 8 of 11 deaths a pathogen could be determined. Deaths were attributable to the following pathogens (# of patients): *K. pneumoniae* (2), *S. suis* (2), *B. pseudomallei*: (1), *N. meningitides* (1), HSV (1) and *E.Coli* (1). Óutcomes with sequelae were caused by: *S. suis* (2), HSV (1) and *S. pneumoniae* (1). Of the 130 patients with clinical outcome, CSF values or a distinct pathogen could be detected in 108 patients with CNS infections (Table 3) Patients in the BI group were significantly older than patients in the VI group (BI: median 54 years, IQR 31.5–62.5, vs VI: median 36.5 years, IQR 28–57, *P* = 0.02). BI patients had significantly high body temperature during their hospital stay (BI: median 39 °C, IQR 39–40, vs VI: median 39 °C, IQR 38–39.5, *P* = 0.004, vs TI: median 38.6 °C, IQR 38.25–39, *P* = 0.03), with quite frequent speech disorders (BI: n = 30 (56%), vs VI: n = 15 (30%), *P* = 0.009, vs TI: n = 0 (0%), *P* = 0.02) and a higher percentage of neck stiffness on clinical examination (BI: n = 53 (98%), vs VI: n = 36 (72%), *P* = 0.0009., vs TI: n = 3 (60%) *P* = 0.002), than TI and VI patients. In contrast, BI cases had a significantly shorter time interval between first reported symptoms and hospital admission compared to VI or TI cases (BI: median 3 days, IQR 2–5, vs. VI: median 4 days, IQR 2–8, *P* = 0.005, vs. TI: median 10 days, IQR 5.5–10, *P* = 0.005). Also, a significant lower GCS was observed in BI cases compared to VI cases (BI: median 13, IQR 12–15, vs VI: median 15, IQR 13–15, *P* = 0.0032). Occurrence of cranial nerve palsy was observed to be significantly higher in the TI than BI cases (BI: n = 1 (2%), vs TI: n = 1 (20%), *P* = 0.028). No significant differences were observed between the three groups with regard to symptoms such as disturbances of consciousness, vomiting, sensory disturbances, headaches and convulsions.

#### Specific risk factors for CNS infections

Of the 112 patients who could be assigned to either the BI, VI or TI group, 16 (13%) had specific risk factors for CNS infections. The most common were diabetes (n = 5; 4%) and previous traumatic brain injury (n = 5; 4%), followed by liver cirrhosis (n = 3; 3%), previous stroke (n = 2; 2%), chronic use of immunosuppressants (n = 2; 2%), chronic alcohol abuse (n = 1; 1%), and 1 patient with bacterial meningitis due to S. suis reported having recently consumed pork and raw pork blood.

## Discussion

For clinicians in low- and middle-income countries (LMICs), making the correct diagnosis and treating patients with CNS infections remains a challenge because they start unnoticed, and the aetiology is unknown. Vietnam is a LMIC acknowledges the burden of death and morbidity due to CNS infections. This is due to the low availability of resources and reduced diagnostic spectrum to identify the factual causes of CNS infections. Our study was conducted from 2014 to 2017 in one of the largest hospitals in Hanoi to obtain data on pathogens causing CNS infections and their clinical course. The pathogen detection rate in our study is comparable to other studies in Southeast Asia. Dependent on the age of patients and the numbers of diagnostic tests, detection rates about 30% to over 50% are achieved^4–7^. Consistent with other reports from this region, we observed bacterial infections, particularly in adults^4,5,7^, with *S. suis* being the most commonly detected pathogen. Although *S. suis* is considered a rare infectious agent of meningitis worldwide, reports of a high incidence of *S. suis* as causative agent of meningitis have been accumulating over the past decade, especially among adults in Southeast Asia^4,5,8^. In particular males are at high risk if they have contact with pigs, e.g. at pig farms or slaughterhouses. High alcohol consumption and dishes with undercooked pork or raw pig blood, called Tiết canh, are also considered as risk factors for *S. suis* infections^8,9^. An associated clinical feature of *S. suis* meningitis is deafness, which was found in four of our *S. suis*-infected patients^8^.

The observed number of *N. meningitidis* cases are consistent with a recent study from Vietnam^7^. A likely reason for the high detection rate of *N. meningitidis* is due to the military environment, where few patients were soldiers housed in communal accommodation or barracks, which is considered a risk factor for *N. meningitidis* infection. Among the viral pathogens tested, HSV 1/2 had contributed to 4 cases with CNS infection. HSV is known to be the most common causative agent for encephalitis with a global incidence up to 4 cases/1,000,000/year^10^. Rapid empirical therapy with acyclovir has been shown to have a significant impact on the neurological outcome of HSV patients^10^. Among the patients examined, 3 rickettsial infections were detected in the CSF, but the CSF was negative for *O. tsutsugamushi*. So far, only few data on rickettsial infections in Vietnam have been published. One study found up to 40% seropositivity for scrub typhus in febrile patients^11^ and another study found a fairly low seroprevalence of about 9% Ig-G antibodies to *Rickettsia* species^12^. Although different approaches were used in the two studies, the data show that the overall epidemiology of rickettsiae in northern Vietnam is not fully understood and further studies are warranted, especially with regard to meningitis caused by rickettsiae as observed in our study.

### Unique CNS infections

Also, a few unique pathogens were detected via blood or CSF culture. For instance, *Aerococcus viridans* was detected in the CSF culture of an 88-year-old female patient. *A. viridans* is a coccus known to cause gaffkaemia, a disease of lobsters, but has also been reported to cause endocarditis, urinary tract infections, arthritis or meningitis in humans^13,14^. This is an interesting finding; however, *A. viridans* cannot be considered a common causative agent of meningitis in adults. Another interesting finding was the detection of *Haemophilus parainfluenzae* in the CSF culture of a 17-year-old male. Although *H. parainfluenzae* is present in the respiratory tract, pathogenicity is considered low and there are reports of respiratory and urinary tract infections as well as endocarditis, bacteraemia and meningitis. Meningitis caused by *H. parainfluenzae* occurs mainly in children or immunocompromised adults^15^.

Also worth mentioning is a case of *Burkholderia pseudomallei* in a 29-year-old male detected in the blood culture and in an abscess formation in the patient's right orbit. The patient died of sepsis and consecutive multiple organ failure. *B. pseudomallei,* a gram-negative, soil dwelling bacterium, was first described in 1927 in Vietnam and causes melioidosis^16^. Infection mainly occurs through percutaneous inoculation or inhalation. Pneumonia and skin infections are the most common clinical manifestations, multiple metastatic abscess formation with sepsis associated complications. Diabetes, alcohol abuse, chronic lung disease and immunosuppression are considered major risk factors for clinical manifestation and poor outcome^17^. Since 1927, *B. pseudomallei* has hardly been recognized and studied as a pathogen in South-East Asian countries, including Vietnam, but increasing cases and awareness have led to additional investigations in the last decade^17^.

In our study, we detected *Stenotrophomonas maltophilia* by blood culture in a 52-year-old man with diabetes and hypertension. Although, this opportunistic infection is difficult to treat, the patient survived the disease episode without sequelae. *S. maltophilia* is a gram-negative multidrug-resistant organism (MDRO) and a globally emerging pathogen that is ubiquitous in aqueous environments and known to form biofilms on biotic and abiotic surfaces. Infections occur as pneumonia, wound or urinary tract infections, meningitis and endocarditis. Known risk factors are prolonged hospitalisation, invasive procedures and mechanical ventilation, indwelling catheters, immunosuppression and cystic fibrosis. *S. maltophilia* can easily be confused with *Pseudomonas* spp. in culture, and thus be a contributor for underreporting or low incidences^18^.

In this study, three patients had coinfections with two different pathogens. While bacterial co-infection could be coincidental, bacterial meningitis and concurrent detection of EBV has been shown to be common and must be considered as reactivation of EBV, especially in immunocompromised individuals, increasing the mortality of affected individuals^19^.

### CNS infections and patient outcome

The significant difference between the CSF values, especially the lymphocyte counts and the CSF glucose levels between the groups BI, VI and TI, is mainly due to the fact that CNS infections without a detectable pathogen were categorized only on the basis of these CSF values. Although the burden of CNS infections in northern Vietnam is well known, there are to date few epidemiological data and data of the clinical course of CNS infections in adults. The data from our study contribute to a clearer picture of the pathogens and their clinical presentation and show that adults are more frequently affected by bacterial pathogens in particular *S. suis*. In terms of clinical outcome, individuals with bacterial (BI) CNS infections had a significantly lower Glasgow Coma Scale, more frequent neck stiffness and speech disorders, and were significantly older with a poor outcome than viral (VI) or tuberculous (TI) CNS infections. Patients with bacterial CNS infections also presented to the hospital ward much earlier after the onset of symptoms, indicating a higher severity of the disease. Additionally, limb paresis was observed only among BI patients. These data indicate that patients with suspected (via CSF values) or proven bacterial CNS infection are at high risk of a poor outcome. This underscores the fact that even if a bacterial CNS infection is suspected, immediate antibiotic therapy should be initiated to prevent severe symptoms and poor outcome.

## Methods

### Study cohort with CNS infections

This prospective hospital-based cohort study was conducted between May 2014 until May 2017 at the Infectious Diseases Department of the 108 Military Central Hospital, a tertiary referral hospital for adults with a catchment area including Hanoi and the northern provinces of Vietnam. The hospital is open to the general public and over 80% of the patients are civilians and 20% of the patients are military personals and their civilian family members. All recruited patients were admitted to the infectious disease ward and were 15 years or older, with suspected meningitis or encephalitis based on CNS infection criteria such as fever, headache, vomiting, neck stiffness, focal neurological signs, seizures and alterations in consciousness, and underwent lumbar puncture^20^. Patients were included in our analysis, if the white blood cell (WBC) count in the CSF was > 10/µl. In 6 patients, complete CSF counts could not be obtained from patient records, but a causative pathogen was found in the CSF. Patients with other aetiologies as a cause for the neurological disorder, and when no informed consent obtainable were excluded. For microbiology cultures and molecular assessment, at least 2 ml of CSF were obtained by lumbar puncture. CSF samples were stored at -80 °C until further use.

A total of 137 CSF samples were investigated. Clinical cases were categorized in three distinct groups by CSF characteristics (turbidity, glucose levels, presence of white blood cells, microbes, or an increase in protein levels) according to international standards^20^. Bacterial CNS infection (BI) defined by WBC > 10 µl, predominance of polymorphonucleocytes, protein > 1 g/l, glucose < 40 mg/dl; aseptic/viral CNS infection (VI) defined by WBC > 10 µl, predominance of lymphocytes, protein < 2 g/l, normal glucose levels; tuberculous CNS infection (TI) defined by WBC > 10 µl, predominance of lymphocytes, protein > 1 g/l, glucose < 40 mg/dl.

Diagnostics and treatment of patients followed the clinical routine. Demographic and clinical data, routine haematology, and clinical biochemistry parameters were recorded for all patients and samples were subjected to blood culture. In addition, CT or MRI imaging was performed. Clinical, demographic and laboratory data was extracted from the hospital records and entered in an electronic data base. The clinical outcome was defined as recovered: no persistent neurological sequelae; partially recovered: persistent neurological disorder and as death.

### Microbiological diagnoses

Microbiological examination was done by microscopy of CSF and by CSF and blood cultures according to the routine standards of the hospital. In brief, two independent venous blood samples of approximately 8 ml were taken from both arms of the patients and 1 ml of CSF from a lumbar puncture. Cultures were performed using the BACTEC™ Plus Aerobic/F System (Becton–Dickinson, Franklin Lakes, NJ, USA). Each positive culture was grown on a selective medium such as blood agar, chocolate agar and MacConkey agar (Merck, Kenilworth, NJ, USA) and incubated in the BD BACTEC™ 9120 device (Becton–Dickinson, Franklin Lakes, NJ, USA) at 36 °C for 18–72 h. When bacterial growth was detected, colonies were selected for species identification using the MALDI-TOF VITEK® MS system for automated microbial identification and antimicrobial susceptibility testing was performed using the automated VITEK® 2 compact system (BioMérieux, Lyon, France).

### Molecular diagnoses

DNA and RNA were extracted from 200 to 140 µl CSF using QIAamp DNA Blood and QIAamp Viral RNA Mini Kit (Qiagen, Hilden, Germany), respectively, following the manufacturer´s instructions. The application of internal controls is critical to overcome false-negative results, either caused because of inhibition or due to human error. Therefore, for each step of nucleic acid testing (NAT), we utilized the bacteriophage MS2 as an internal control, when molecular assays were performed. PCR was performed on a thermal cycler (Eppendorf, Hamburg, Germany) and/or Real-Time PCR was performed on a LightCycler 480 Instrument II (Roche, Basel, Switzerland) targeting 19 distinct pathogens. PCR-based assays were carried out for detection of *Staphylococcus aureus, Pseudomonas aeruginosa, Klebsiella pneumoniae*; whereas real-time PCR were carried out for the detection of Herpes simplex virus *1/2,* Cytomegalovirus, Epstein-Barr virus, Parvovirus B19*,* Dengue virus, Measles virus, *Orientia tsutsugamushi*, Rickettsiae, *Mycobacterium tuberculosis, Neisseria meningitidis, Streptococcus pneumoniae, Streptococcus suis, Cryptococcus neoformans, Haemophilus influenza b, Listeria monocytogenes,* and *Leptospira interrogans.* The details of the primers and probes used for PCR and real-time PCR, respectively, and the conditions for thermal cycling are summarized in the supplementary data (Table S1).

### Statistical analysis

Statistical analysis was performed via JMP® (SAS Institute, North Carolina, USA) and the significance level was set at *p* < 0.05. Quantitative parameters of laboratory values are given as median with quartile ranges where appropriate. Statistical analysis was performed by non-parametric Mann–Whitney–Wilcoxon rank sum test for continuous variables and Chi square test for categorical data.

### Ethics approval

Informed written consent was obtained after a detailed explanation of the study at the time of blood and CSF sampling from all hospitalized patients and/or relatives and from the parents if subjects were < 18 years old. The study protocol was approved by the institutional Review Board of the 108 Military Central Hospital, Hanoi, Vietnam (108MCH/RES/MENTNGITIS-V-D3-25042017). All experiments were performed in accordance with ICH-GCP guidelines and regulations.

## Supplementary Information

Supplementary Information.

## Data Availability

All data relevant to the study are included in the article or uploaded as supplementary information.
