# Supplementary material for: Aetiologies and clinical presentation of central nervous system infections in Vietnamese patients: a prospective study
Source: Sci Rep. 2022 Oct 27;12:18065. doi: 10.1038/s41598-022-23007-0 (PMC9613671; doi:10.1038/s41598-022-23007-0)
Supplement: Supplementary file 1 — Supplementary Information. [file 41598_2022_23007_MOESM1_ESM.docx]

**Supplementary table S1:** Details of primers and probes utilized in the study for diagnosing CNS infections

| Primers and Probes | Cycling conditions | Reference |
| --- | --- | --- |
| ***Herpes simplex* ½ (real time-PCR**) | 95°C, 3 min.  40x (95°C, 5sec.; 60°C, 30 sec.) | Unpublished data |
| HSV.108-F1: GTCATCCAGATMTCCTGTCTGC |  |  |
| HSV.108-R1: CCGAGCGAAAACAGGAGGA |  |  |
| HSV.108-Pro1: FAM-CTACGACCTGTCCACCACCGCCC-1BQF |  |  |
| ***Mycobacterium tuberculosis* (real time-PCR**) | 95°C,15 min.;  40x (95°C, 15 sec.; 60°C, 30 sec.) | [1] |
| MTB-F: GCCGGATCAGCGATCGT |  |  |
| MTB-R: GCAAAGTGTGGCTAACCCTGAA |  |  |
| MTB-P: FAM-TTCGACGGTGCATCT-MGB |  |  |
| ***Orientia tsutsugamushi*** **(real time-PCR**) | 95°C,15 min.;  40x (95°C, 15 sec.; 60°C, 60 sec.) | Unpublished data |
| O.tsu108S-F3: CAGCAAGCWCAAGCTACAG |  |  |
| O.tsu108S-R3: CGCTGCAATTTAACAAGATC |  |  |
| O.tsu108S-Pro: FAM-CAAGAAGCAGYAGCAGCWGCAGC-1QBH |  |  |
| ***Rickettsia genus* (real time-PCR**) | 95°C, 15 min.;  40x (95°C, 15 sec.; 60°C, 60 sec.) | Unpublished data |
| Rick.S-CS-F1: ATACCGTCGCAAATGTTYAC |  |  |
| Rick.S-CS-R1: GTCTTCRTGCATTTCTTTCC |  |  |
| Rick.S-CS-Pro1: FAM-TGTGCCATCCAGCCTAYGGTTCTTGC-1QBH |  |  |
| **Cytomegalovirus (real time-PCR)** | 95°C, 2 min.;  40x (95°C, 10 sec.; 60°C, 50 sec.) | [2] |
| CMV-Set4-F: ACGATTCACGGAGCACCAG |  |  |
| CMV-Set4-R : GCTGACGCGTTTGGTCAT |  |  |
| CMV-Set4/12 Pro: FAM-CAGGCGGATCACCACGTTCG-TAMRA |  |  |
| **Epstein-Barr-Virus (real time-PCR)** | 95°C, 2 min.;40x  (95°C, 10 sec.; 60°C, 50 sec.) | [3] |
| TS-EBV-Fwd1: CCCAACACTCCACCACACC |  |  |
| TS-EBV-Rev1: TCTTAGGAGCTGTCCGAGGG |  |  |
| TS-EBV-Pro1: FAM-CACACACTACACACACCCACCCGTCTC-Iowa Black FQ |  |  |
| ***N. meningitides* (real time-PCR)** | 95°C, 5min.;  40x (95°C, 10 sec.;60°C, 20 sec.) | [4] |
| N.mening. F: TGTGTTCCGCTATACGCCATT |  |  |
| N.mening R: GCCATATTCACACGATATACC |  |  |
| N. mening. Probe: FAM-AACCTTGAGCAA"T"CCATTTATCCTGACGTTCT-SpC6; BHQ1- the quenchers are internally placed at the “T” base region |  |  |
| ***S. pneumoniae*** **(real time-PCR)** | 95°C, 5min.;  40x (95°C,10 sec.; 60°C, 20 sec.) | [5] |
| S. pneu F373 CDC: ACGCAATCTAGCAGATGAAGCA |  |  |
| S. pneu R424: TCGTGCGTTTTAATTCCAGCT  S. pneu. Probe: FAM-TGCCGAAAACGC"T"TGATACAGGGAG -SpC6  BHQ1 - the quenchers are internally placed at the “T” base region |  |  |
| ***P. aeruginosa* (PCR)** | 95°C, 2min.;  40x (95°C, 30 sec.; 58°C, 30 sec.; 72°C, 30 sec.)  72°C, 7 min.; 4°C | [6] |
| P. aer. F: GGGGGATCTTCGGACCTCA |  |  |
| P. aer. R: TCCTTAGAGTGCCCACCCG |  |  |
| ***S.suis*** **(real time-PCR)** | 95°C, 5 min.,  40x (95°C, 10 sec., 60°C, 20 sec.) | [7] |
| S.suis F: GGTTACTTGCTACTTTTGATGGAAATT |  |  |
| S.suis R: CGCACCTCTTTTATCTCTTCCAA |  |  |
| S.suis probe: FAM-TCAAGAATCTGAGCTGCAAAAGTGTCAAATTGA-TAMRA |  |  |
| ***Cryptococcus neoformans* (real time-PCR)** | Cryptococcus neoformans, RT- PCR KIT, Product code: PKIT11015PCRmax LTD., UK |  |
| ***Haemophilus influenza* (real time-PCR)** | 95°C, 5 min.;  40x (95°C, 10 sec.; 60°C, 20 sec.) | [8] |
| H. influenz F: GGTTAAATATGCCGATGGTGTTG |  |  |
| H. influenz R: TGCATCTTTACGCACGGTGTA |  |  |
| H. influenz Probe: FAM -TTGTGTACACTCCGT"T"GGTAAAAGAACTTGCAC- SpC6  BHQ1 - the quenchers are internally placed at the “T” base region |  |  |
| ***Leptospira interrogans* (PCR)** | 1x94°C,3min.;  40x(94°C,30sec.; 50°C,30sec.; 72°C,60 sec.), 72°C, 10 min.; 4°C | [9] |
| LFB1-F: CATTCATGTTTCGAATCATTTCAAA |  |  |
| LFB1-R: GGCCCAAGTTCCTTCTAAAAG |  |  |
| Listeria monocytogens **(real time -PCR)** | 95°C, 5 min.;  40x (95°C, 10 sec.; 60°C, 20 sec.) | [10] |
| Lis.monF: TTTCATCCATGGCACCACC |  |  |
| Lis.monR: ATCCGCGTGTTTCTTTTCGA |  |  |
| Lis.monProb: FAM-CGCCTGCAAGTCCTAAGACGCCA-TAMRA |  |  |
| **S. aureus (PCR)** | 1x94°C,3min.;  40x(94°C,30sec.; 50°C,30sec.; 72°C,60 sec.), 72°C, 10 min.; 4°C | [11] |
| SA442 F1: gtcgggtacacgatattcttcacg |  |  |
| SA442 R1: ctctcgtatgaccagcttcggtac |  |  |
| **K. pneumoniae (PCR)** | 1x94°C,3min.;  40x(94°C,30sec.; 57°C,30sec.; 72°C,60 sec.), 72°C, 10 min.; 4°C | [12] |
| K. pneu. F: ATTTGAAGAGGTTGCAAACGAT |  |  |
| K. pneu. R: TTCACTCTGAAGTTTTCTTGTGTTC |  |  |
| **Measles (real time-PCR)** | 95°C, 2 min.;  40x (95°C, 10 sec.; 60°C, 20 sec.) | [13] |
| MV N3F: TGGCATCTGAACTCGGTATCAC |  |  |
| MV N3R: TGTCCTCAGTAGTATGCATTGCAA |  |  |
| MV N3Probe: FAM-CCGAGGATGCAAGGCTTGTTTCAGA- BHQ1 |  |  |
| **Dengue-Serotype 1-4 (DENV-1-4) multiplex (real time -PCR)** | 95°C, 2 min.;  40x (95°C, 10 sec.; 62°C, 60 sec.) | Unpublished data |
| DEN1-4_F1: ACM GCH TGG GAY TTY GGW TC |  |  |
| DEN1-4_R1: CCR CTK CCA CAT TTB ARY TCT |  |  |
| DENV1 probe: FAM-GTTCAGCGGTGTTTCCTGGACCAT-BHQ1 |  |  |
| DENV2 probe 1: HEX-GCTCTCCACCAAGTTTTYGGAGCAAT-BHQ1 |  |  |
| DENV2 probe 2: HEX-CTGCCTTYAGTGGGGTTTCATGGACYA-BHQ1 |  |  |
| DENV3 probe: Cy5-CACAGCCCTGTTTAGYGGAGTCTCATG-BHQ3 |  |  |
| DENV4 probe: ROX-ATGTTTGGAGGRGTYTCATGGATG-BHQ2 |  |  |
| **Parvovirus B19 (real time-PCR)** | 50°C, 2 min; 95°C, 15 min.;  40x (95°C, 15 sec.; 60°C, 60 sec.) | [14] |
| PVB19-Taq1: TTTCAAAGTCATGGACAGTTATCTGA |  |  |
| PVB19-Taq2: TTGTGTAAGTCTTCACTAGATAATACTGCATT |  |  |
| TaqMan: FAM-CCTTATCATCCAGTAGCAGTCATGCAGAACCT-TAMRA |  |  |

**References:**

[1] E.T. Leung, L. Zheng, R.Y. Wong, E.W. Chan, T.K. Au, R.C. Chan, G. Lui, N. Lee, M. Ip, Rapid and simultaneous detection of Mycobacterium tuberculosis complex and Beijing/W genotype in sputum by an optimized DNA extraction protocol and a novel multiplex real-time PCR, J Clin Microbiol 49(7) (2011) 2509-15.

[2] L.A. Verkruyse, G.A. Storch, S.M. Devine, J.F. Dipersio, R. Vij, Once daily ganciclovir as initial pre-emptive therapy delayed until threshold CMV load > or =10000 copies/ml: a safe and effective strategy for allogeneic stem cell transplant patients, Bone Marrow Transplant 37(1) (2006) 51-6.

[3] Y.M. Lo, L.Y. Chan, K.W. Lo, S.F. Leung, J. Zhang, A.T. Chan, J.C. Lee, N.M. Hjelm, P.J. Johnson, D.P. Huang, Quantitative analysis of cell-free Epstein-Barr virus DNA in plasma of patients with nasopharyngeal carcinoma, Cancer Res 59(6) (1999) 1188-91.

[4] J. Khumalo, M. Nicol, D. Hardie, R. Muloiwa, P. Mteshana, C. Bamford, Diagnostic accuracy of two multiplex real-time polymerase chain reaction assays for the diagnosis of meningitis in children in a resource-limited setting, PLoS One 12(3) (2017) e0173948.

[5] G. Carvalho Mda, M.L. Tondella, K. McCaustland, L. Weidlich, L. McGee, L.W. Mayer, A. Steigerwalt, M. Whaley, R.R. Facklam, B. Fields, G. Carlone, E.W. Ades, R. Dagan, J.S. Sampson, Evaluation and improvement of real-time PCR assays targeting lytA, ply, and psaA genes for detection of pneumococcal DNA, J Clin Microbiol 45(8) (2007) 2460-6.

[6] T. Spilker, T. Coenye, P. Vandamme, J.J. LiPuma, PCR-based assay for differentiation of Pseudomonas aeruginosa from other Pseudomonas species recovered from cystic fibrosis patients, J Clin Microbiol 42(5) (2004) 2074-9.

[7] N.T. Mai, N.T. Hoa, T.V. Nga, D. Linh le, T.T. Chau, D.X. Sinh, N.H. Phu, L.V. Chuong, T.S. Diep, J. Campbell, H.D. Nghia, T.N. Minh, N.V. Chau, M.D. de Jong, N.T. Chinh, T.T. Hien, J. Farrar, C. Schultsz, Streptococcus suis meningitis in adults in Vietnam, Clin Infect Dis 46(5) (2008) 659-67.

[8] CDC, Chapter 10: PCR for Detection and Characterization of Bacterial Meningitis Pathogens: Neisseria meningitidis, Haemophilus influenzae, and Streptococcus pneumoniae; <https://www.cdc.gov/meningitis/lab-manual/chpt10-pcr.html>, (2016).

[9] P. Bourhy, S. Bremont, F. Zinini, C. Giry, M. Picardeau, Comparison of real-time PCR assays for detection of pathogenic Leptospira spp. in blood and identification of variations in target sequences, J Clin Microbiol 49(6) (2011) 2154-60.

[10] A. Le Monnier, E. Abachin, J.L. Beretti, P. Berche, S. Kayal, Diagnosis of Listeria monocytogenes meningoencephalitis by real-time PCR for the hly gene, J Clin Microbiol 49(11) (2011) 3917-23.

[11] U. Reischl, H.J. Linde, M. Metz, B. Leppmeier, N. Lehn, Rapid identification of methicillin-resistant Staphylococcus aureus and simultaneous species confirmation using real-time fluorescence PCR, J Clin Microbiol 38(6) (2000) 2429-33.

[12] Y. Liu, C. Liu, W. Zheng, X. Zhang, J. Yu, Q. Gao, Y. Hou, X. Huang, PCR detection of Klebsiella pneumoniae in infant formula based on 16S-23S internal transcribed spacer, Int J Food Microbiol 125(3) (2008) 230-5.

[13] K.B. Hummel, L. Lowe, W.J. Bellini, P.A. Rota, Development of quantitative gene-specific real-time RT-PCR assays for the detection of measles virus in clinical specimens, J Virol Methods 132(1-2) (2006) 166-73.

[14] B.D. Bultmann, K. Klingel, K. Sotlar, C.T. Bock, H.A. Baba, M. Sauter, R. Kandolf, Fatal parvovirus B19-associated myocarditis clinically mimicking ischemic heart disease: an endothelial cell-mediated disease, Hum Pathol 34(1) (2003) 92-5.
